# Lifestyle risk factors of white matter brain aging: Evidence, potential mechanisms, and future direction

**DOI:** 10.4103/NRR.NRR-D-25-01039

**Published:** 2026-01-02

**Authors:** Tianzhou Ma, Zhenyao Ye, Li Feng, Shuo Chen

**Affiliations:** Department of Epidemiology and Biostatistics, School of Public Health, University of Maryland, College Park, MD, USA; Maryland Psychiatric Research Center, Department of Psychiatry, School of Medicine, University of Maryland, Baltimore, MD, USA; Department of Epidemiology and Public Health, School of Medicine, University of Maryland, Baltimore, MD, USA

Population aging has greatly increased the global burden of neurodegenerative diseases. As we age, the structure and function of our brain undergo significant changes, including brain volume decline, cortical thinning, white matter deterioration, and altered functional connectivity, among others, resulting in cognitive decline and an elevated risk of neurodegenerative diseases. However, there is significant individual variation in how fast the brain ages and the timing of when these changes emerge and progress into age-related diseases. A previous study has used advanced machine learning (ML) methods to predict age from magnetic resonance imaging (MRI) data in healthy people (Franke and Gaser, 2019). Various risk factors, both modifiable and non-modifiable, were found to influence the brain aging process, either delaying or accelerating its progression (Cole, 2020).

In this article, we focused on studies of white matter (WM) brain aging and their lifestyle risk factors, inspired by recent findings that brain cognitive function is largely determined by WM integrity. We reviewed and summarized studies that utilize ML models to predict WM brain age from MRI data and identify lifestyle risk factors for WM brain aging, and discussed plausible mechanisms. This perspective further emphasized that integrating multi-omics and multimodal imaging data represents essential next steps for future brain aging studies, enabling a comprehensive understanding of how lifestyle factors influence the aging brain.

**Individual variation in white matter brain aging:** WM microstructures of the brain, particularly the condition of myelin and axonal fibers, which reflects the integrity and organization of WM tracts, are known to deteriorate with age and can be effectively assessed by the diffusion tensor imaging (DTI) technique. As WM microstructures are sensitive to subtle changes and early aging, they are often used to predict brain age in ML models. Multiple regional DTI metrics, including fractional anisotropy, axial diffusivity, mean diffusivity, and radial diffusivity, have been used to predict white matter brain age.

**Figure 1:**
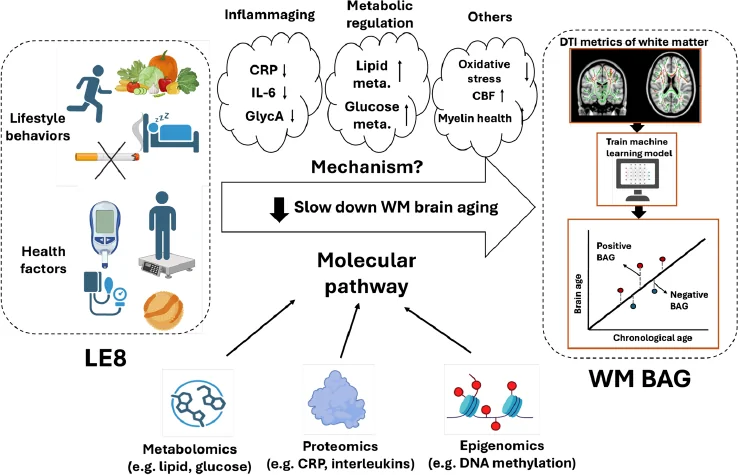
Healthy lifestyle (high LE8 scores) slows down white matter brain aging: Understanding the mechanisms and molecular pathways. LE8 comprises eight key metrics representing lifestyle behaviors (diet, physical activity, smoking, and sleep) and health factors (body mass index, lipids, HbA1c, and blood pressure). The machine learning model takes DTI metrics of WM (e.g., fractional anisotropy measures) as input to predict chronological age and output brain age. WM BAG, calculated as the difference between predicted brain age and chronological age, was used as the outcome in the study. Different hypotheses have been proposed on the potential mediating mechanisms, which include reducing the levels of inflammaging biomarkers (e.g., CRP, IL-6, and GlycA), improving glucose and lipid metabolism, or reducing oxidative stress, enhancing CBF, and promoting myelin repair. In future studies, the collection of multi-omics data, including but not limited to metabolomics, proteomics, and epigenomics data, will help understand the molecular pathways by which healthy lifestyles and potential intervention strategies may delay brain aging. Created with BioRender.com. BAG: Brain Age Gap; CBF: cerebral blood flow; CRP: C-reactive protein; GlycA: glycoprotein acetylation; IL: interleukin; LE8: Life’s Essential 8; meta.: metabolism; WM: white matter.

Groundbreaking consortium projects such as the Human Connectome Project and the UK Biobank have made vast amounts of individual neuroimaging data accessible and created an unprecedented opportunity for brain age research. The brain age prediction model is first trained using neuroimaging data from a large sample of healthy individuals, and then tested in new individuals to generate their predicted brain age. The neuroimaging data need to undergo rigorous preprocessing steps to derive meaningful features related to aging, e.g., fractional anisotropy measures for each WM tract that quantify the level of diffusion anisotropy and reflect WM integrity. These derived neuroimaging features are used as predictors in a regression model with chronological age as the outcome to predict brain age. Brain Age Gap is calculated by subtracting chronological age from predicted brain age. Positive Brain Age Gap is considered a marker of accelerated aging, associated with poorer brain health and a higher likelihood of cognitive decline and dementia, while negative Brain Age Gap indicates delayed brain aging and implies overall better brain health (**Figure 1**). Due to the high-dimensionality and complex correlation between neuroimaging features, advanced ML models are often used. Gradient boosting, random forest, regularized regression, and deep learning methods are among the commonly used methods to predict WM brain age from DTI data. Individual differences in WM brain aging have been identified, highlighting substantial variability in the rate and extent of age-related WM microstructural changes. These individual discrepancies in WM brain aging can be partially explained by a range of genetic and environmental factors and is associated with markers of physiological aging and cognitive aging, and risk of neurodegenerative disease (Cole, 2020).

**Healthy lifestyle can revert the white matter aging process:** A number of modifiable risk factors, related to individual behaviors and lifestyle choices, were found to be associated with WM brain aging in healthy people. Our previous studies found tobacco smoking accelerated WM brain aging by 0.16 years for every extra cigarette consumed per day (Mo et al., 2022), high blood pressure accelerated WM brain aging by 0.37 years for every 10 mmHg increase in blood pressure especially among late middle-aged women (Feng et al., 2023). Other factors, including alcohol consumption and diet, were also found to be associated with WM structural change and brain aging (Daviet et al., 2022; Feng et al., 2024).

Most existing studies usually evaluate the impact of individual lifestyle risk factors in isolation. The American Heart Association introduced Life’s Essential 8 (LE8) as a comprehensive set of eight metrics of health behaviors that collectively support cardiovascular health. We evaluated the effect of the composite LE8 score (0–100) on WM brain aging and found that adherence to healthy lifestyle (i.e., higher LE8 score), including a favorable diet, no smoking, higher level of physical activity, sufficient sleeping, favorable body mass index, and normal blood pressure and HbA1c levels, can delay WM brain aging regardless of gender, age and genetically susceptible (e.g., Apolipoprotein E ε4 [*APOE4*] carriers *versus* non-carriers) groups (**Figure 1**; Feng et al., 2025b). On average, the brain will be 113 days younger per 10-point increase in LE8 score. This finding, as a summary of individual lifestyle factor findings from previous studies, has emphasized the importance of overall healthy lifestyle and behavior in slowing down WM brain aging to maintain a younger brain and reduce the risk of early-onset neurodegenerative disease. This may also suggest public health preventative strategy that underscores a broader usage of lifestyle interventions in promoting brain health.

In addition to the overall effect, it is interesting to note that there exists some gender and age discrepancy in the effect of lifestyle factors on brain aging. For example, the effect of blood pressure on brain aging was found to be most prominent among late middle-aged women mainly possibly due to the fluctuation of sex hormones among women of this age group. It is also found that the *APOE4*-lifestyle interaction on brain aging is more dominant in women 40–49 and 60–69-year groups, suggesting age-dependent shifts in *APOE4*-related inflammatory and metabolic pathways. These factors need to be taken into account while designing lifestyle related preventive strategies and public health interventions to slow down brain aging and cognitive decline.

**Gaining the mechanistic understanding of healthy lifestyle-delayed brain aging process:** Given the consistent findings of effect of lifestyle factors on WM brain aging, it is natural and of clinical interest to ask how a healthy lifestyle maintains the integrity of white matter and slows down brain aging, i.e., what is the underlying biological mechanism (**Figure 1**).

Different hypotheses considering the process have been proposed: (1) healthy lifestyle can reduce aging-related inflammation (a.k.a. inflammaging), which contributes to white matter degradation. Part of the hypothesis has been supported by our recent study that allostatic load (the cumulative “wear and tear” on the body caused by chronic stress) which includes inflammatory biomarker C-reactive protein as a key component, has negative impact on WM brain aging (Feng et al., 2025a). Longitudinal studies can be conducted to test the potential mediating roles of inflammaging biomarkers and track changes in lifestyle, inflammaging biomarkers and WM structural change over time; (2) healthy lifestyle can improve glucose and lipid metabolism in the brain, which is known to induce neuroinflammation and disrupt WM microstructure. In the other study, we tested and found several plasma low-density lipoprotein markers in the blood from nuclear magnetic resonance data are associated with WM integrity from UK Biobank (Ye et al., 2024), partially supporting the role of metabolic regulation, where lifestyle factors. Future studies could design trials that use positron emission tomography scans to trace the glucose and lipid metabolism in the brain using different radioactive tracers (e.g., ^18^F-FDG) and study their temporal change in response to lifestyle intervention; (3) healthy lifestyle can also enhance cerebral blood flow, support oligodendrocyte function, promote myelin repair, and reduce oxidative stress. These effects may collectively contribute to maintaining WM integrity and preventing age-related neurodegenerative changes. Several advanced neuroimaging techniques, such as arterial spin labeling MRI, myelin water imaging, magnetic resonance spectroscopy, and positron emission tomography, can be employed to capture these physiological and microstructural changes in the brain.

**Future direction: Incorporation of multi-omics and multimodal imaging data:** The advancement of technologies in recent decades has enabled the collection of molecular data of the same subjects across multiple biological layers, collectively termed multi-omics data, including genomic, epigenomic, transcriptomic, proteomic, and metabolic data (**Figure 1**). The advent of these rich omics data equipped with advanced statistical and ML tools have provided an unprecedented opportunity for us to study the biological mechanism and molecular pathway by which healthy lifestyles and potential intervention strategies may delay brain aging. For example, it is critical to analyze proteomic data related to C-reactive protein, cytokines, interleukins, glycoproteins and pro-inflammatory transcription factors to understand the role of inflammation in the lifestyle-brain aging pathway. Likewise, the inclusion of metabolic data including key lipid and glucose markers will help understand how healthy lifestyle modulates their metabolic pathways, which in turn slows brain aging, and the inclusion of epigenomic data, such as DNA methylation, helps us understand the cumulative effects of lifestyle and other environmental factors on the brain. While large biobanks such as UK Biobank and All of Us have provided extensive genetic, phenotypic and electronic health record data, the availability of multi-omics data remains limited. There is a pressing need for the incorporation of multi-omics data in brain aging research for their promising utility and growing potential.

Conversely, the brain aging process is rather complex, with WM microstructural change representing only one aspect of neurobiological alterations. For example, different from a universal structural decline of the brain during aging, using resting-state functional connectivity data, we only observed partial functional decline associated with aging, primarily in sensorimotor, dorsal/ventral attention regions, and basal ganglia regions (Pan et al., 2024). In addition, the trajectory of human brain aging is nonlinear, with distinct rates of change occurring at different stages of life (Di Biase et al., 2023). A multimodality approach that integrates structural (diffusion) and functional (resting-state and task-based) MRI data has the benefit of providing a richer and more comprehensive understanding of the individual differences in brain aging (Cole, 2020).

*This work was supported by the National Institute on Drug Abuse (NIDA) of National Institute of Health under the award number 1DP1DA048968-01 to SC and TM, by the National Institute on Drug Abuse (NIDA) under the award number 1K01DA059603-01A1 to TM, by the University of Maryland Grand Challenge Grant, MPower Brain Health and Human Performance seed grant and EPIB Department Pilot Award to TM.*
